# Supplementary figures and images for: A mathematical model explores the contributions of bending and stretching forces to shoot gravitropism in Arabidopsis
Source: Quant Plant Biol. 2020 Dec 15;1:e4. doi: 10.1017/qpb.2020.5 (PMC10095965; doi:10.1017/qpb.2020.5)

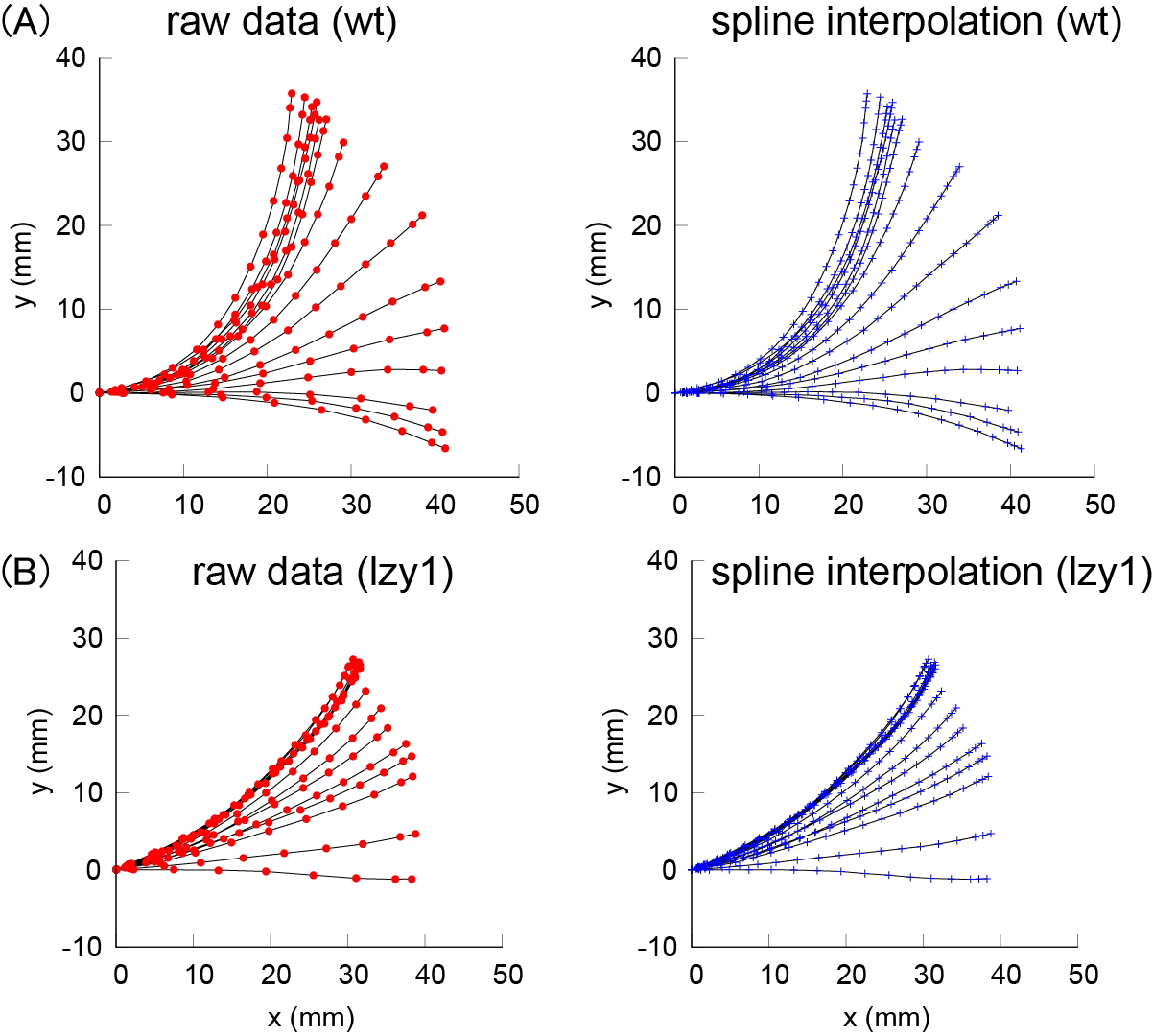

Supplement: Supplementary file 1 [file S2632882820000053sup001.zip › S2632882820000053sup001.jpg]

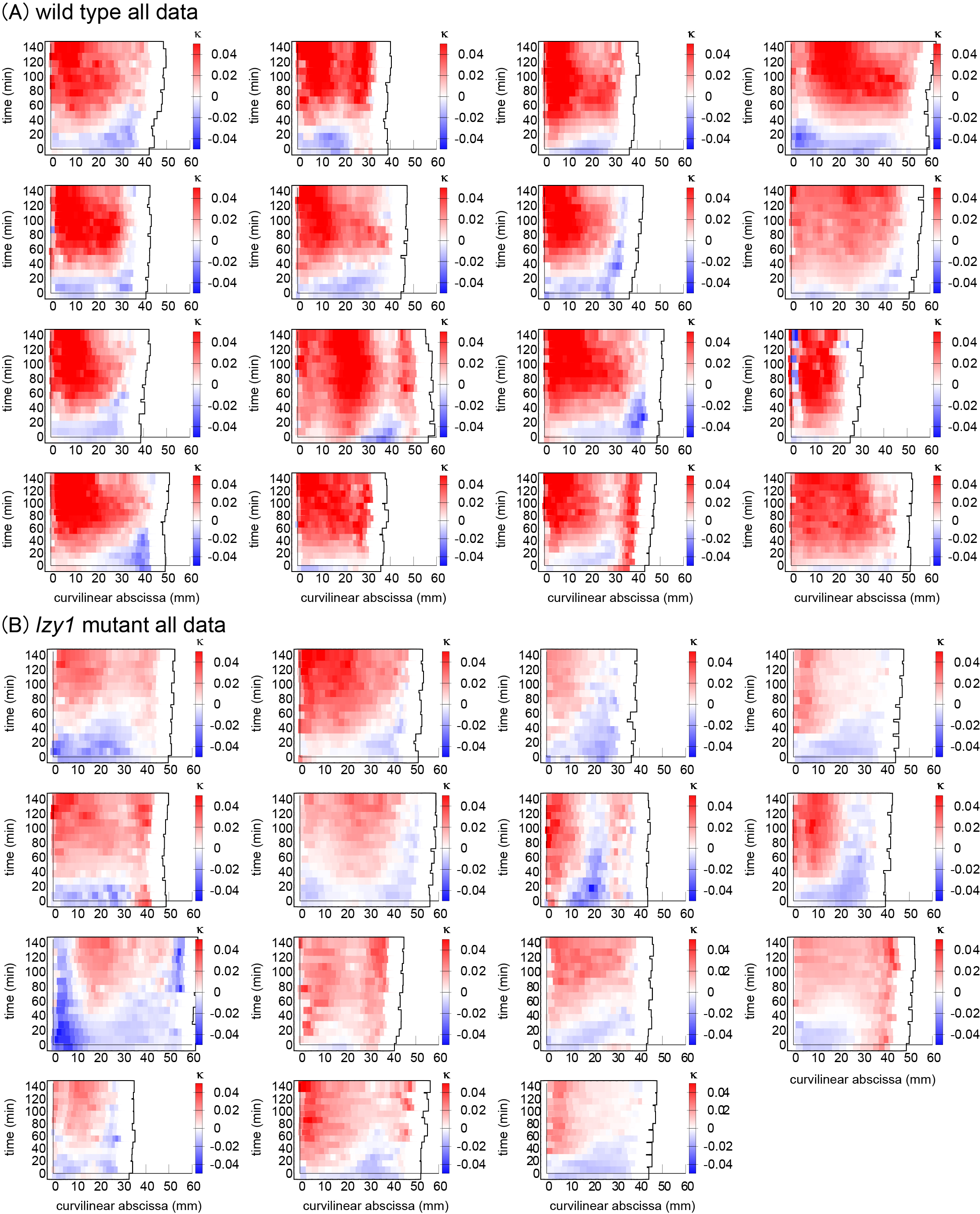

Supplement: Supplementary file 1 [file S2632882820000053sup001.zip › S2632882820000053sup002.jpg]

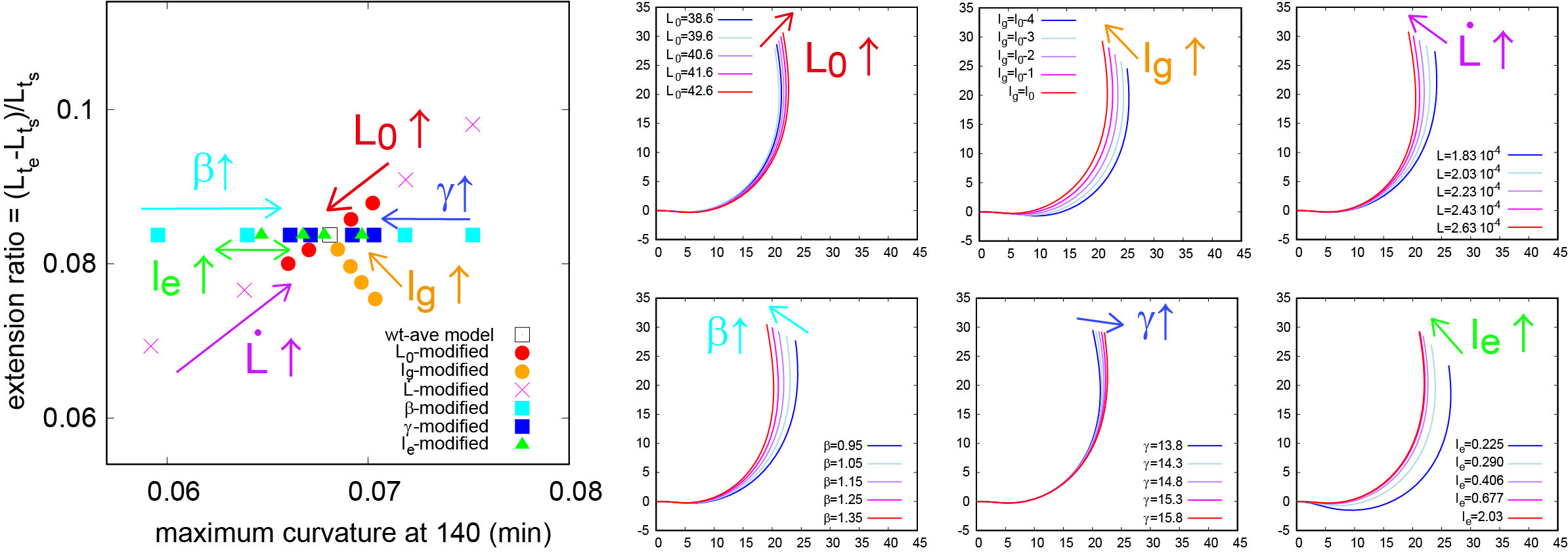

Supplement: Supplementary file 1 [file S2632882820000053sup001.zip › S2632882820000053sup003.jpg]

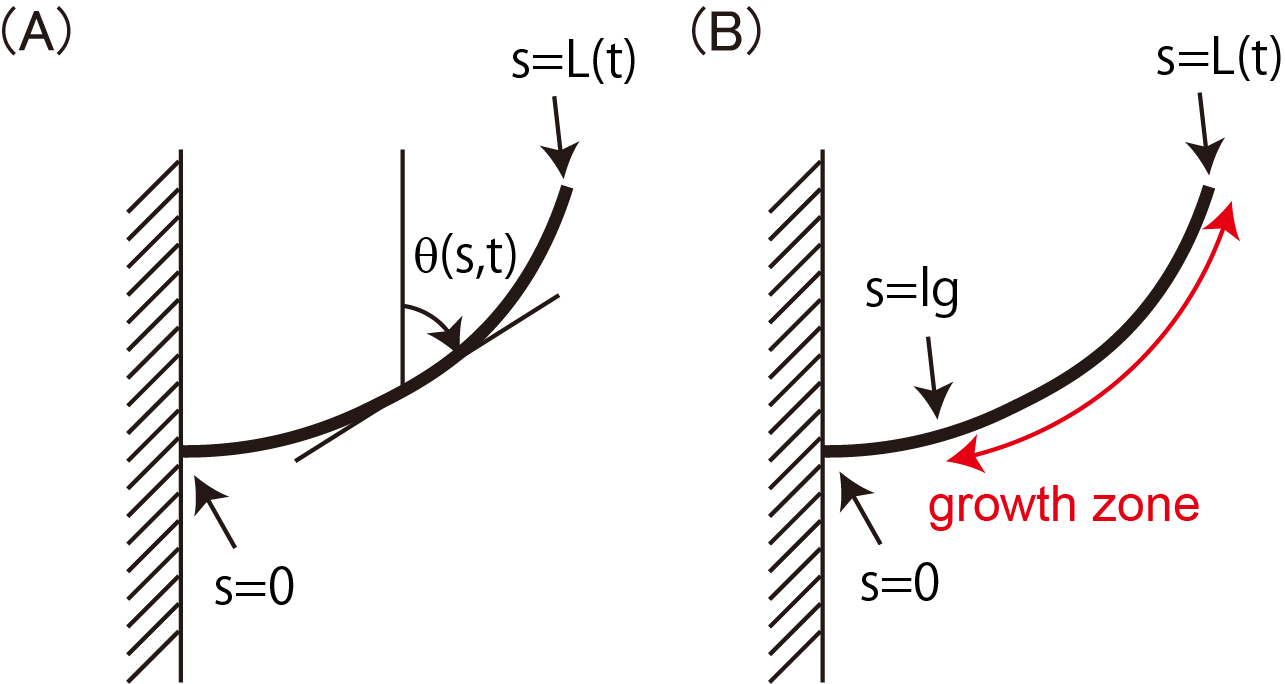

Supplement: Supplementary file 1 [file S2632882820000053sup001.zip › S2632882820000053sup004.jpg]

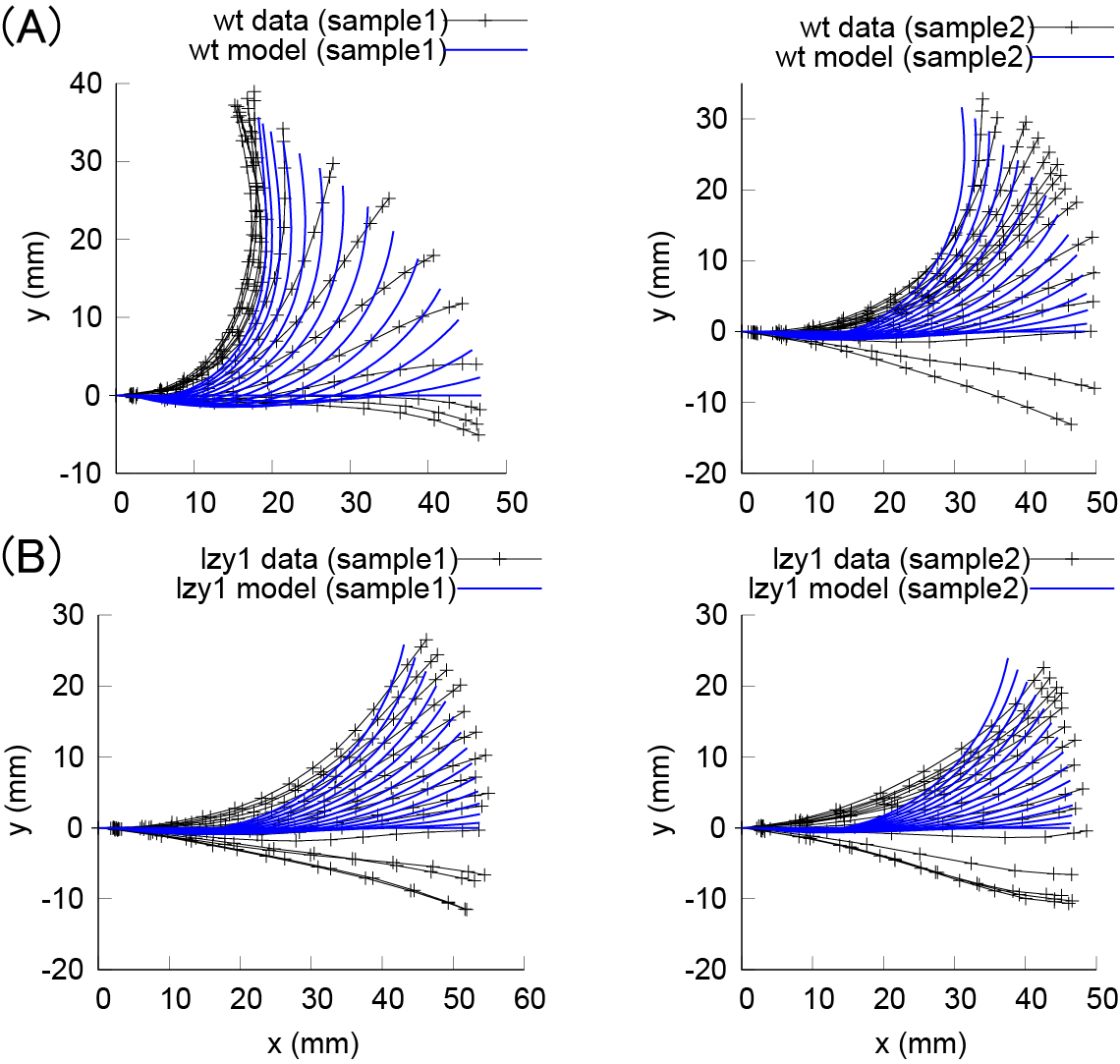

Supplement: Supplementary file 1 [file S2632882820000053sup001.zip › S2632882820000053sup005.jpg]

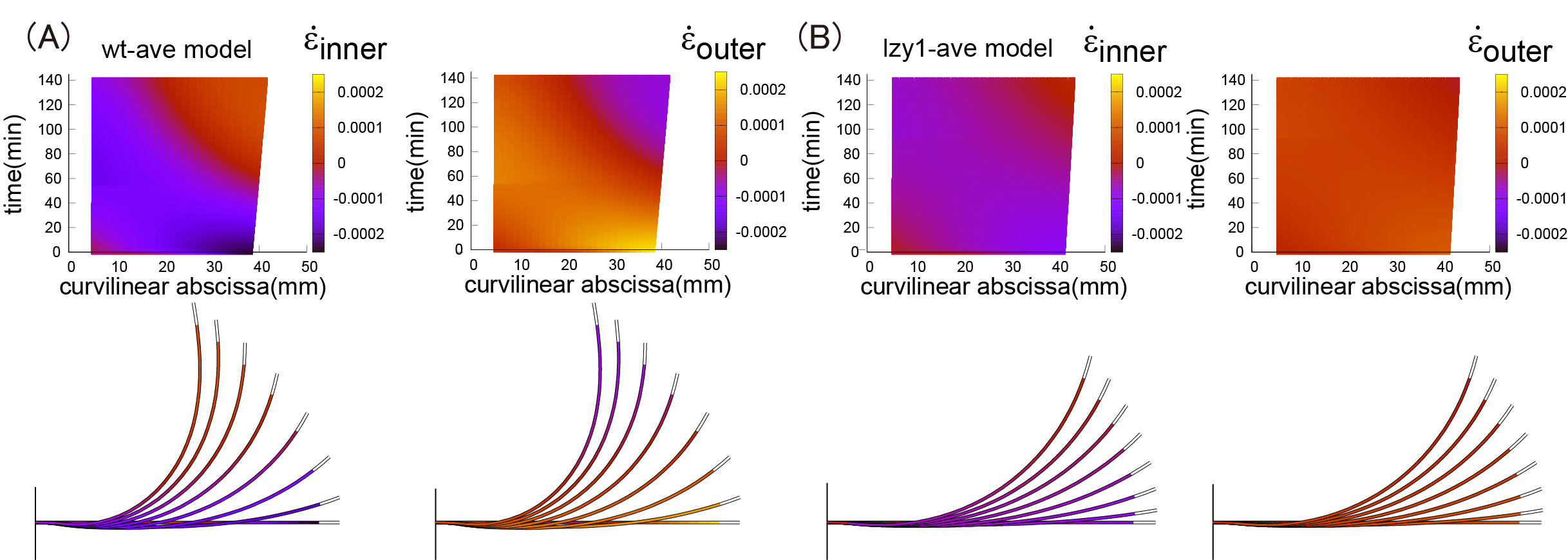

Supplement: Supplementary file 1 [file S2632882820000053sup001.zip › S2632882820000053sup006.jpg]

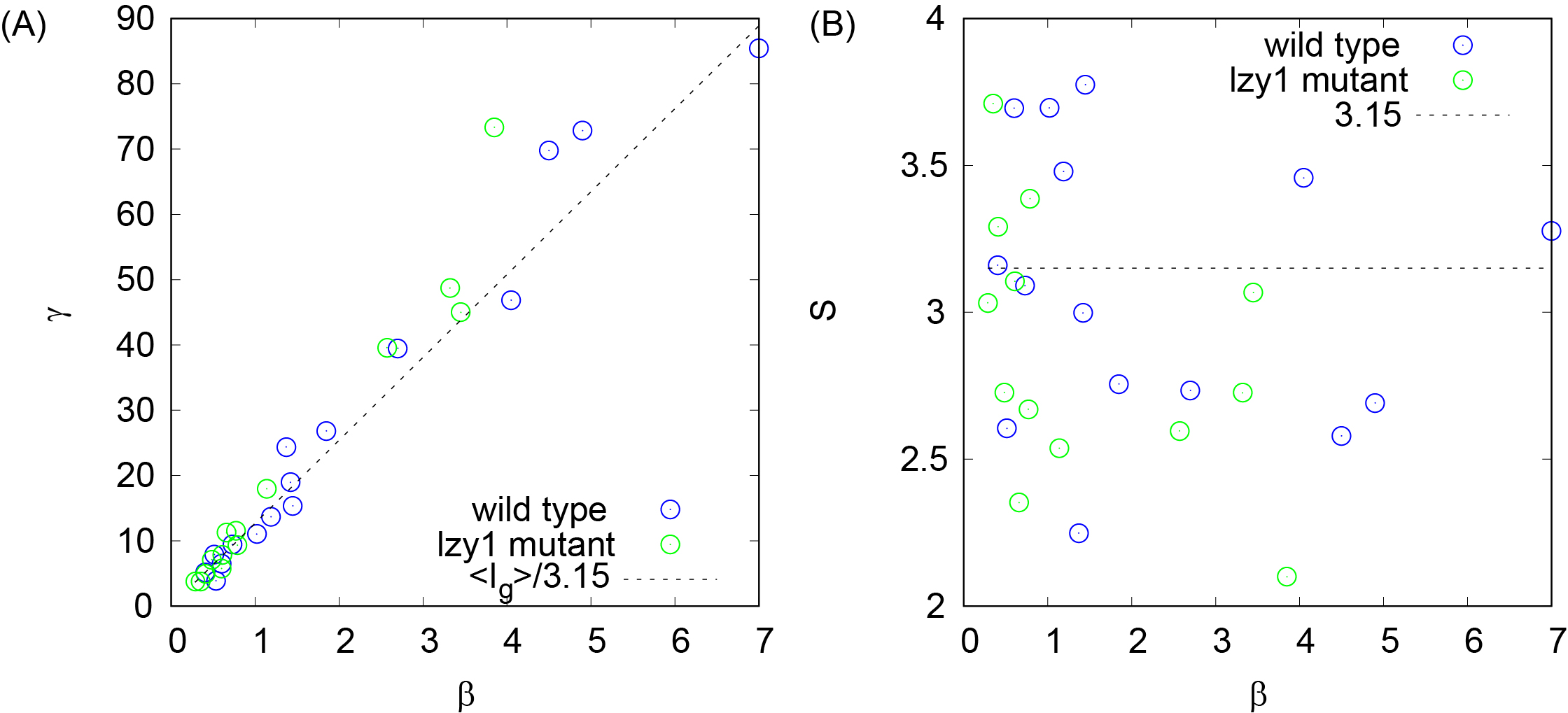

Supplement: Supplementary file 1 [file S2632882820000053sup001.zip › S2632882820000053sup007.jpg]
